# Delay discounting predicts COVID-19 vaccine booster willingness

**DOI:** 10.1186/s41235-024-00609-y

**Published:** 2025-01-23

**Authors:** Julia G. Halilova, Samuel Fynes‑Clinton, Caitlin M. Terao, Donna Rose Addis, R. Shayna Rosenbaum

**Affiliations:** 1 Department of Psychology and Centre for Integrative and Applied Neuroscience, York University, 4700 Keele St., Toronto, ON M3J 1P3 Canada; 2 Baycrest Academy for Research and Education, Toronto, Canada; 3 Department of Psychology, University of Toronto, Toronto, Canada; 4 School of Psychology, The University of Auckland, Auckland, New Zealand

**Keywords:** Decision-making, Public health measures, Vaccine hesitancy, Immunity maintenance

## Abstract

Developing ways to predict and encourage vaccine booster uptake are necessary for durable immunity responses. In a multi-nation sample, recruited in June–August 2021, we assessed delay discounting (one’s tendency to choose smaller immediate rewards over larger future rewards), COVID-19 vaccination status, demographics, and distress level. Participants who reported being vaccinated were invited back one year later (n = 2547) to report their willingness to receive a booster dose, along with reasons for their decision. After controlling for demographic variables and distress level, a greater tendency to discount future rewards was associated with reduced willingness to receive a booster dose. Thematic coding revealed that the most common reason for booster willingness was protection against COVID-19, and for unwillingness was non-necessity. The results identify delay discounting as a behavioral predictor of booster willingness that may be used to inform tailored approaches to increase booster uptake (e.g., trust in science vs. vaccine mandates).

**Supplementary Information:**

The online version contains supplementary material available at 10.1186/s41235-024-00609-y.

## Introduction

In the wake of the COVID-19 pandemic that swept the world, and with new variants and other infectious diseases on the horizon, it is imperative to reflect on lessons learned during COVID-19 to prepare for future public health crises. Behavioral science has a critical role to play in this endeavor, as the decisions and behaviors of individuals determine the success of many public health strategies. Throughout the course of the COVID-19 pandemic, recommended protective behaviors have undergone dynamic shifts in response to the evolving landscape of the crisis. The early response leaned heavily on government mandates, compelling individuals to adhere to public health measures, such as physical distancing, mask-wearing, and stringent hand hygiene, to be able to engage in social interactions, work, and travel. As vaccines were subsequently developed and rolled out, the focus pivoted toward promoting widespread vaccination to achieve population-level immunity, again often with the use of mandates. With the pandemic having transitioned from an acute emergency to a managed state, booster doses are recommended to sustain immunity over time, but individuals now have more autonomy over the decision to receive booster doses. This decision also needs to be made repeatedly, with the roll out of new boosters annually. Continuous vaccination plays a pivotal role in preventing the resurgence and rapid spread of infectious diseases by maintaining widespread immunity within communities (Greenwood, 2014) and limiting pathogen transmission (Feldman et al., 2021; Greenwood, 2014; Pollard & Bijker, 2021). Thus, understanding the determinants that shape the decision to receive additional booster doses is of crucial importance to encourage sustained maintenance of public health in the long run.

Worldwide, willingness to receive vaccine boosters has been consistently lower than that for initial vaccination doses, with this disparity becoming more pronounced over time (Dziedzic et al., 2022; Lazarus et al., 2023; Limbu & Huhmann, 2023; Lin et al., 2023Young et al., 2024) . This discrepancy raises questions about the distinct nature of decision-making between the primary and booster vaccine doses. With the continued emergence of new COVID-19 variants (e.g., BA2.86, EG.5), it is particularly important to ensure sustained public immunity (Lazarus et al., 2023) by maximizing the effectiveness of public health strategies targeting vaccine booster behavior. However, vaccine hesitancy is a complex issue influenced by various factors, including cognitive biases, social norms, and economic considerations. Previous research has shown that COVID-19 vaccine booster hesitancy is predicted by gender (female), age (younger), education level and socio-economic status (lower; Noh et al., 2022; Paul & Fancourt, 2022), health status (those without pre-existing health conditions; Paul & Fancourt, 2022), pregnancy (Attia et al., 2022), history of previous COVID-19 infection (Attia et al., 2022), low level of worry about COVID-19 (Paul & Fancourt, 2022), as well as perceived risks, restricted or no benefits, and vaccination barriers (Qin et al., 2022).

Although the utility of behavioral economic measures in public policy, particularly within the context of public health initiatives, has been supported in the literature (e.g., Matjasko et al., 2016; Ruggeri, 2022a; van Bevel et al., 2020), the role of behavioral economic measures in predicting vaccine booster hesitancy remains underexplored. The current research contributes to the literature on booster hesitancy by investigating the role of delay discounting, a behavioral economic measure of one’s tendency to favor smaller immediate rewards over larger later rewards (Green & Myerson, 2004). Delay discounting has been shown to be a reliable predictor of engagement in a wide range of health-related behaviors, including healthy dietary habits (Appelhans et al., 2019), physical exercise (Albelwi et al., 2019), smoking cessation (e.g., Baker et al., 2003; Bickel et al., 1999), substance use (MacKillop et al., 2011), and more recently, the decision to receive an initial vaccination against SARS-CoV-2 (Halilova et al., 2022; Hudson et al., 2023). Importantly, delay discounting is a modifiable trait variable (Odum, 2011), which makes it a target mechanism of public health interventions with a variety of potential health benefits. For example, previous research has shown that cueing individuals to imagine future, personally relevant, specific events leads to a reduced rate of discounting of delayed rewards (Bromberg et al., 2017; Ciaramelli et al., 2021; Mok et al., 2020; Rung & Madden, 2018). In the realm of delay discounting related to vaccination, understanding the reward context (i.e., reasons for vaccine booster willingness and hesitancy) is crucial for examining how individuals weigh immediate versus delayed rewards when making decisions about vaccination. Insights into delay discounting patterns and reasons for vaccine booster willingness may inform public health policies (e.g., campaigns that appeal to public trust versus implementing mandates; Goldenberg et al., 2023) to promote vaccine booster willingness.

In this longitudinal study, we test the potential utility of delay discounting among those who received the main dose of the COVID-19 vaccine as a marker of their booster vaccine hesitancy a year later. Guided by previous findings showing an association between delay discounting and vaccine hesitancy of the main COVID-19 dose (Halilova et al., 2022, 2024a), we made a priori hypotheses about the relationship between delay discounting and booster willingness before collecting Time 2 data. Specifically, we predicted that delay discounting would be a significant predictor of booster willingness after controlling for demographic and distress variables—important predictors of main dose vaccination decisions (Halilova et al., 2022, 2024a). We controlled for factors that have previously been shown to predict vaccination status, including age (e.g., Guay et al., 2022), education level (Guay et al., 2022; Noh et al., 2022), relative income level (Larkin, 2022; Paul & Fancourt, 2022), essential worker status (Lavoie et al., 2022), psychological distress (Penner et al., 2023), and intolerance of uncertainty (Brun et al., 2022). We also assessed reward context related to vaccine booster willingness and hesitancy by asking participants to describe reasons for their decisions.

## Method

### Participants

Participants were recruited through an online platform (Prolific.co) between June, 2021 and August 2021 to participate in Part 1 of a larger longitudinal study (Halilova et al., 2022, 2024a). Using Prolific’s built-in inclusion/exclusion filters, the study was available only to Prolific users meeting the following inclusion criteria: aged 18 years or older, fluent in English, normally residing in one of 13 target countries (United States, Canada, Mexico, United Kingdom, Italy, France, Portugal, the Netherlands, Spain, Germany, Poland, Australia, and New Zealand) across North America, Europe, Australasia, and free from neurological impairments or learning disabilities. Approximately one year later, participants who completed Part 1 were invited to participate in Part 2 between June 28, 2022 and August 28, 2022 (see Fig. S1 for the data collection flowchart). A total of 3,185 individuals completed both Part 1 and Part 2 of the study. As this study focuses on booster hesitancy in individuals who have received the main dose of the COVID-19 vaccine, the 270 participants who reported being unvaccinated in Part 2 were excluded, as were the 368 who did not provide a response to the question about vaccine boosters (the outcome variable of interest).

The final data set was composed of 2,547 participants who were on average 30.92 (*sd* = 11.42) years old; 1417 were female, 1075 male, 51 non-binary, and 4 preferred not to respond. Approximately 30% of the sample had achieved secondary level education, 49% had an undergraduate degree, and 20% of the sample achieved postgraduate education. Approximately 22% of the sample self-identified as essential workers, working in occupations supplying critical services during the pandemic: government; health and safety (e.g., healthcare, emergency response); utilities (e.g., water, energy, sanitation, transport, communications); food (e.g., supermarkets); and manufacturing. Given the international nature of our sample, we used a subjective measure of relative income where participants estimated their current income relative to others in their own country/region on a sliding scale with three anchors (0 = low, 50 = average, 100 = high; Adler et al., 2000; Smith et al., 2019); the average relative income was 38.83 (*sd* = 23.8). Approximately 19% of the sample (484 participants) indicated during Part 2 that they would choose not to receive a vaccine booster dose, if one was recommended.

The study was approved by the York University and Baycrest Research Ethics Boards (REB# 19–07) for research with human participants, and all research was conducted in accordance with the Declaration of Helsinki. All the participants provided informed online consent prior to their participation in the study.

### Measures

#### Vaccination status and booster willingness

First, we identified those participants who, at the time of completing Part 2, had received at least one dose of the COVID-19 vaccine. Similar to the measure of vaccination status used in Part 1 of the study (see Halilova et al., 2022, 2024a), participants chose between 5 options to indicate their vaccination status: 1 = yes, I have received all necessary doses; 2 = yes, although I require another dose; 3 = no, but I am planning to get vaccinated; 4 = no, I am not planning to get vaccinated; or 5 = prefer not to say. A binary *vaccination status* variable was created, distinguishing between those who were vaccinated (fully or partially) or not (including both those who were planning and not planning to get vaccinated in the future). Those participants indicating they had received at least one dose (i.e., options 1 or 2) were then asked the following question to assess booster willingness: *“If another dose of the COVID-19 vaccine was recommended, would you choose to receive it?”* Participants responded using the following scale: 0 = no, 1 = yes, or 2 = prefer not to say.

#### Reasons for booster willingness and hesitancy

Participants had the opportunity to explain their reason(s) for their choice about willingness to get a vaccine booster dose if one was recommended. Participant responses were scored according to the primary reason described, using the categories of vaccination reasons developed by Halilova et al. (2024b): (1) ending or containing the pandemic (e.g., “it would help fight the pandemic”), (2) protecting oneself or others from COVID-19 (e.g., “to be safe and keep others safe as well”), (3) (non-necessity (e.g., “for regulatory and travel purposes”), (4) (mis)trust (in science, government, or vaccines; e.g., “not enough evidence that vaccines are safe”), (5) health reasons (e.g., “medical complications”, “bad side effects from the vaccinations”), or (6) other (e.g., “I am afraid of needles”). To establish inter-rater reliability, S.F.C. and two independent raters (W.F. and R.T.) scored mentions of these reasons in 100 randomly-selected participant responses (responses could have more than one reason). Raters had 86–87% agreement with S.F.C. on their categorizations of reasons and acceptable inter-rater reliability (Cohen’s Kappa = 0.92–0.93). The two independent raters then scored all of the responses from the participants reported on in this paper.

#### Delay discounting task

In this intertemporal choice paradigm (Ciaramelli et al., 2021; Halilova et al., 2022; Mok et al., 2020), participants were presented with pairs of monetary values and tasked with deciding between hypothetical immediate, smaller rewards that changed from trial to trial, and a larger, later reward of $2,000. Each participant had to make six decisions at seven distinct temporal delays for the larger reward (1 week, 1 month, 3 months, 6 months, 1 year, 3 years, and 10 years before receiving the $2000 reward). The process employed an iterative approach in which the value of the immediate reward was modified based on the participant’s prior decision at a particular delay. This adjustment aimed to converge on an immediate reward value equivalent in subjective worth to the delayed reward. The initial adjustment was set at half the difference between the immediate and delayed amounts offered in the initial trial. Subsequent adjustments were consistently half of the preceding values. For instance, in a scenario where a future $2000 reward could be obtained in 3 years, the participant’s initial choice would be between “Receive $1000 now or $2000 in 3 years.” Should the participant select “$2000 in 3 years,” the subsequent choice would be between “$1500 now or $2000 in 3 years.” If the participant then opted for “$1500 now,” the subsequent choice would present “$1250 now or $2000 in 3 years.” After the completion of the sixth and final trial for each condition, the subjective value of the delayed reward was estimated as the immediate reward amount for a hypothetical seventh trial. A higher subjective value for the delayed reward indicated a lesser discounting rate, while a lower value represents a greater tendency for short-sighted decision-making. The degree of discounting was quantified by analyzing the subjective values of the rewards across the seven delays and calculating the Area-under-the-Curve (AuC), which is a single, neutral measure of discounting (Myerson et al., 2001). The discounting measure of AuC also has the benefit of generating approximately normally distributed scores (Myerson et al., 2001). These scores range from 0 to 1, where a lower AuC value represents a more pronounced discounting rate (i.e., greater tendency for short-sighted decision-making). Given that delay discounting is a relatively stable individual characteristic (Odum, 2011), discounting scores obtained in Part 1 of the study were used as predictors of booster willingness in Part 2 of the study.

#### Psychological Distress Index

Presence and severity of anxiety and depressive symptoms were assessed with the Generalized Anxiety Disorder 7-item (GAD-7) scale (Spitzer et al., 2006) and the Patient Health Questionnaire 9-item (PHQ-9) scale (Kroenke et al., 2001), respectively. Participants rated the frequency of symptoms experienced over the past two weeks on a four-point scale (0 = not at all; 3 = nearly every day). For each scale, a total score was computed, where higher scores reflect more severe symptoms. Total scores from these measures were standardized and then summed to create a *psychological distress index*.

#### Intolerance of Uncertainty Scale (IUS-12)

The IUS-12 is a 12-item measure of one’s difficulties tolerating uncertainty (Carleton et al., 2007). Participants used a 6-point scale (0 = not at all characteristic of me; 5 = entirely characteristic of me) to respond to items measuring two factors of intolerance of uncertainty: prospective anxiety, the cognitive component of intolerance of uncertainty that indicates one’s tendency to worry about future events (e.g., “I always want to know what the future has in store for me”) and inhibitory anxiety, the behavioral component of intolerance of uncertainty that represents avoidance tendencies in the face of uncertainty (e.g., “I must get away from all uncertain situations”; Carleton et al., 2007). *Intolerance of uncertainty* score was calculated as a sum of participants’ responses to IUS-12, ranging from 0 to 60.

#### Attention checks

To identify random responders, three items from the Conscientious Responder Scale (Marjanovic et al., 2014) were included at select points within the survey in Part 1 (e.g., “To answer this question, please choose option three, neither agree nor disagree.”). In Part 2, only one item was included given that this survey was much shorter. None of the participants in the current subsample failed the attention check.

### Procedure

Data were collected on the above measures using two online Qualtrics surveys, as part of a larger longitudinal study. During Part 1 (June – August 2021), among other measures, participants provided informed consent, demographic information, and completed the GAD-7, PHQ-9, IUS-12, the delay discounting task, as well as indicated their COVID-19 vaccination status. During Part 2 (June – August 2022), among other measures (see Table S1), participants responded to a series of COVID-related questions, including their vaccination status, as well as their willingness to get a vaccine booster dose if one was recommended.

## Results

### Booster willingness

Approximately 81% of the sample (2063 participants) indicated that they were willing to receive a vaccine booster dose. A multilevel logistic regression model was constructed using R packages *lme4* (Bates et al., 2012) and *lmerTest* (Kuznetsova et al., 2017) with booster willingness at *Time 2* (no vs. yes) as the outcome variable, and age, education level, relative income, essential workers status, psychological distress, intolerance of uncertainty, and delay discounting as predictors. Each participant’s booster willingness (Level 1) was nested within country (Level 2) to account for possible systematic differences across countries. A likelihood ratio test showed that the model accounted for significantly more variance in the data compared to an unconditional intercept-only model, *χ*^2^*(7)* = 25.60, *p* =  < 0.001.

**Fig. 1:**
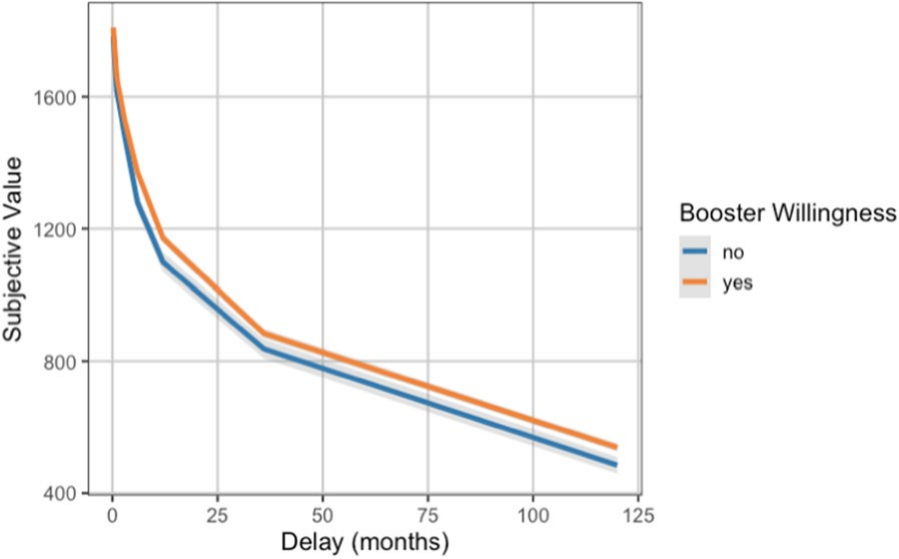
Discounting curves in those willing and unwilling to receive a booster dose. Subjective value (mean indifference point) of the $2,000 delayed reward as a function of the delay to its receipt. Area-under-the-Curve (AuC) was used as the measure of delay discounting. Individuals discounting the future rewards more steeply (i.e., those with smaller AuCs, shown in blue) were not willing to receive a booster dose. Shaded areas represent standard errors around the curves

The tendency to choose larger future rewards over smaller immediate ones, represented by greater AuC, significantly increased the odds of being willing to get a booster vaccine dose after controlling for other variables in the model (*OR* = 1.92, *p* = 0.002; Table 1; Fig. 1).

**Table 1:** Results of the multilevel logistic regression model predicting booster willingness

Fixed effects	*b*	*SE*	*z*	*p*	OR	95% CI
Intercept	0.95	0.35	2.69	0.007	2.59	[1.29, 5.16]
Age^†^	0.01	0.01	1.63	0.103	1.01	[0.99, 1.02]
Gender	−0.23	0.11	−2.15	0.032	0.79	[0.64, 0.98]
Education level	0.02	0.08	0.32	0.750	1.03	[0.88, 1.19]
Relative income^†^	−0.14	0.06	−2.41	0.016	0.87	[0.78, 0.98]
Essential worker status	−0.08	0.13	−0.66	0.507	0.92	[0.72, 1.18]
Psychological distress^†^	0.08	0.03	2.40	0.017	1.08	[1.02, 1.16]
Intolerance of uncertainty^†^	−0.08	0.06	−1.39	0.164	0.92	[0.82, 1.04]
Delay discounting (AuC)	0.67	0.21	3.13	0.002	1.95	[1.28, 2.96]
Random Effects	Estimate	*SD*				
Intercept error variance (country)	0.17	0.41				

AuC = Area-under-the-Curve. CI = Confidence interval; OR = odds ratio; SD = standard deviation; SE = standard error of the mean

^†^The variable was scaled to improve model fit

Among control variables in the model, only relative income and psychological distress were significant predictors of willingness to get a vaccine booster dose if one was recommended (*p* < 0.05; Table 1). Controlling for other variables in the model, individuals who rated themselves higher on the measure of relative income were less likely to be willing to get a vaccine booster dose if one was recommended, *OR* = 0.86, 95% CI [0.77, 0.96]. Controlling for other variables in the model, individuals who reported experiencing more psychological distress were more likely to be willing to get a vaccine booster dose if one was recommended, *OR* = 1.10, 95% CI [1.03, 1.17]. An additional analysis that controls for other variables measured at Time 2 is presented in Table S2. Even with these additional controls, delay discounting remained a significant predictor of booster willingness.

### Reasons for booster willingness and hesitancy

In Part 2, 2536 participants provided reasons why they would (*n* = 2054) or would not (*n* = 482) be willing to receive a vaccine booster dose if one was recommended. Of those willing to receive a vaccine booster, 59% reported protection against COVID-19 as their reason (40% for themselves, 5% for their families, and 14% for others). 26% reported that their booster willingness was based on trust (12% trust in science, 10% in vaccines, and 4% in government/authorities). Additional reasons for a willingness to get a booster included necessity (7%, e.g., for social, work or ethical reasons), ending or containing the pandemic (3%), health reasons (1%), and “other” responses which did not belong to any aforementioned category (4%; e.g., “Why not”).

Of the 482 participants who indicated they were unwilling to receive a vaccine booster, 24% based their decision on mistrust (18% in vaccines, 1% in science and 1.5% government/authorities, with 3.5% reporting a general mistrust). Other reported reasons included non-necessity (53%), adverse health risks (15%), and having sufficient current protection (2.49%), and “other” responses (5%; e.g., “I see no reason to take it”).

## Discussion

In the current research, we investigated delay discounting as a predictor of COVID-19 vaccine booster willingness. The finding that individuals who are better able to delay gratification (i.e., engage in less delay discounting) are more willing to get vaccinated with a booster dose if one is recommended is consistent with previous literature on delay discounting and engagement in health-related behaviors (e.g., Daugherty & Brase, 2010; Robles et al., 2011), including vaccines (e.g., Freitas-Lemos et al., 2023; Halilova et al., 2022). Importantly, we found that delay discounting was a significant predictor of willingness to get a vaccine booster dose, even after statistically accounting for the effects of demographic and emotional factors that have previously been shown to predict vaccine hesitancy.

The current findings enhance our understanding of the decision-making process related to COVID-19 booster vaccine doses and suggest that in order to maintain long-term population immunity against the virus, strategies should focus on encouraging people to think more about the delayed benefits of vaccination. The reasons provided by participants for their vaccine booster decisions after receiving at least one main dose of the vaccine also offer interesting insights into potential mechanisms contributing to these decisions, and suggest ways that public health campaigns and educational initiatives could be curated to bolster effectiveness. It might be necessary to consider two broad categories of interventions: one type that emphasizes the reasons mentioned by the participants who expressed *willingness* to receive a vaccine booster dose and another type that deemphasizes or resolves the concerns mentioned by participants *unwilling* to receive a booster. The majority of those in our sample who were willing expressed the desire to protect themselves and others from COVID-19 as the primary reason for willingness to get a vaccine booster dose, which is consistent with previous research showing the role of empathy and social norms in vaccination (Drążkowski et al., 2022).

In addition to advancing knowledge of the predictors of willingness to get boosters, our results offer insights into why some people are unwilling to receive vaccine boosters against COVID-19. Over 50% of our participants who decided against receiving a booster dose explained that they believed it was unnecessary. This finding aligns with Sinclair et al. (2023c), who reported that many individuals in the U.S. identified lack of awareness about eligibility or availability, as well as overconfidence in their immunity, as primary reasons for their unwillingness to receive the bivalent booster dose. Given the known risks to health from a SARS-CoV-2 infection (e.g., Del Rio et al., 2020; Khaswal et al., 2022), this finding suggests that biased risk perception is a factor in some people’s unwillingness to receive boosters, and may in part underlie our finding that short-sighted thinking evident on the delay discounting task also predicted unwillingness. It is notable that the current research was conducted in individuals who were already vaccinated with at least one main dose of the vaccine, suggesting potential discrepancies in the perceived risk or benefits of the main doses versus booster doses of the COVID-19 vaccine. From a public health perspective, this observation suggests that, in addition to emphasizing social norms, it may also be necessary to address the biased perception of risk (e.g., Byerley et al., 2024; Nuñez Sahr et al., 2024; Sinclair et al., 2023a). For example, the fact that the majority of people believe that vaccine boosters are necessary and that boosters will protect them and others from the virus in the long-term could be highlighted.

Examining ways of correcting biased risk perception in the context of long-term maintenance of immunity against infectious diseases is an important avenue of future public health research rooted in psychological science (e.g., Sinclair et al., 2023b). For example, the deliberation process about receiving a booster vaccine likely involves weighing the more immediate risks of booster side effects with the relatively delayed health risks posed by the possibility of being infected by the virus. Indeed, Limbu and Huhmann’s (2023) framework for booster vaccine hesitancy posits that booster complacency reflects a lack of concern about COVID-19, biased perception of the health risks associated with contracting COVID-19, as well as the perceived benefits and efficacy of boosters. Delay discounting likely contributes to booster complacency given that one’s tendency to discount the future probably influences risk perception when it comes to COVID-19 and vaccination (Jiang & Dai, 2021). Taken together with the current findings, it suggests that public health campaigns may benefit from presenting information about COVID-19 and benefits of vaccination in a way that corrects for the biased perception of risk captured by cognitive phenomena like delay discounting.

Mistrust in the COVID-19 vaccine was another common reason mentioned by approximately 25% of those unwilling to receive a booster, even after having received at least one main dose. This finding suggests the need to improve the public’s faith in vaccines, particularly once vaccines become non-mandated. In times of rapid mobilization, like the COVID-19 pandemic, direct interventions like government mandates (e.g., lockdowns and travel restrictions) that bypass cognitive and decision-making processes prove most effective in eliciting short-term behavioral changes (Brewer et al., 2017; Broomell & Chapman, 2021). However, it is possible that despite such short-term effectiveness, the rapid and forceful introduction of the COVID-19 vaccine came with unanticipated long-term negative effects (Dube et al., 2022; Goldenberg et al., 2023). Feeling forced into getting vaccinated without being able to deliberate and make one’s own informed decisions could lead people to lose trust in government and consequently reduce their willingness to comply with future policies and recommendations to receive booster doses (Goldenberg et al., 2023). Additional longitudinal research investigating the unintended consequences of various public health interventions may provide insights to guide future applications and effectiveness of various interventions. It is possible that for sustained uptake of protective behaviors, interventions that consider intrinsic motivations and thoughts (e.g., risk beliefs, vaccine efficacy confidence) as well as social factors (e.g., norms, altruism) may be most effective (Broomell & Chapman, 2021). This highlights the need to adjust the initial rollout of vaccination campaigns to focus more on instilling confidence in vaccines, as well as balancing mandates with a sense of autonomy, giving people space to make their own informed decisions without feeling forced to get vaccinated.

### Limitations

There are several important limitations to consider. Although the current study was conducted in a multinational sample, the observed results may not generalize beyond the thirteen Western, educated, industrialized, rich, and democractic (WEIRD) countries included in the final sample. Our understanding of booster willingness would benefit from future work replicating the findings in non-WEIRD countries, given the well-established association between delay discounting and economic inequality (Ruggeri et al., 2022b).

It is possible that the association between delay discounting and booster willingness may be attenuated by environmental factors, including number of cases and hospitalizations, media attention, as well as government and institutional policies. It would be important to replicate these findings in different contexts, including during times of low rates of COVID-19 exposure, the emergence of new strains of COVID-19, and the re-emergence of other infectious diseases (e.g., influenza, measles).

Although it is possible that general cognitive abilities contributed to vaccination decision-making, we did not include direct measures of cognitive function in the current study. The relationship between delay discounting and cognitive abilities is complex and not fully understood, with some studies suggesting only weak associations, especially with intellectual function and cognitive control (e.g., Yeh et al., 2021). Due to time constraints and the limitations of self-report measures, we focused on demographic variables (e.g., income, education) that are known to influence both delay discounting and vaccine hesitancy. Future research could benefit from more comprehensive assessments of cognitive functioning to better understand its potential role in vaccine decision-making.

Another remaining generalizability question relates to the use of a monetary paradigm to measure delay discounting. Investigating the effects of non-monetary measures of delay discounting (e.g., social discounting; Jones & Rachlin, 2009) on booster willingness would serve to improve our understanding of the association between the two. Considering the steeper discounting rate of non-monetary rewards compared to the monetary ones (Baker et al., 2003; Odum et al., 2020), we might expect an even stronger association between non-monetary measures of delay discounting and booster willingness.

The analyses presented in this research are correlational in nature, not allowing us to confirm a causal relationship between delay discounting and booster willingness. Future research should focus on establishing a causal relationship between these variables by testing the effect of delay discounting interventions (e.g., imagining personally relevant future events) on booster willingness.

### Future directions

Our findings have important implications for future research on booster hesitancy interventions. Future research on interventions for booster hesitancy would benefit from testing the effectiveness of combining societal concerns with strategies aimed at reducing delay discounting and promoting long-term thinking. For instance, emphasizing the longer-term benefits of booster vaccination in protecting not only the individual but also the broader community, thereby increasing the salience of delayed societal rewards, could be particularly effective. Existing interventions that cue individuals to imagine engaging in personally relevant future events to reduce delay discounting (Ciaramelli et al., 2021; Mok et al., 2020) could be modified to have people imagine collective future events facilitated by the enhanced protection afforded by boosters. Our study indicates that future research focused on mechanisms and mediation models should also investigate the reasons *why* people engage in these protective health behaviors so that public health campaigns can be better contextualized and target individuals’ primary reasons for vaccination (un)willingness.

## Conclusion

Overall, this research shows that of individuals who had received at least one dose of the COVID-19 vaccine, those who are better able to delay gratification (i.e., opting for larger later rewards over smaller immediate rewards) are more likely to accept COVID-19 vaccine booster doses if they are recommended. The results suggest that successful promotion of long-term immunity may require greater emphasis on protecting the health of the community, instilling trust in vaccines and government, increasing sense of autonomy when making decisions, and correcting biased risk perception and short-sighted thinking.

## Supplementary Information

Additional file 1

## Data Availability

Anonymized, raw, and cleaned data have been deposited in a public repository hosted by the Open Science Framework https://osf.io/z932y/?view_only=87123c53ebbf470082f24bdf6d7ad557. The materials used in this study are either described in the method section or publicly available.

## Abbreviations

AuC: Area-under-the-Curve
IUS-12: Intolerance of Uncertainty Scale-12
