# Supplementary material for: Delay discounting predicts COVID-19 vaccine booster willingness
Source: Cogn Res Princ Implic. 2025 Jan 23;10:1. doi: 10.1186/s41235-024-00609-y (PMC11757841; doi:10.1186/s41235-024-00609-y)
Supplement: Supplementary file 1 — Additional file 1 [file 41235_2024_609_MOESM1_ESM.docx]

**Supplementary Materials**

**S1. Supplemental Methods**

**Figure S1. Data Collection Flow Chart**

**Table S1. Complete List of Variables Collected at Times 1 and 2**

| **Time 1**  **(June – August, 2021)** | | | **Time 2**  **(June – August, 2022)** | | |
| --- | --- | --- | --- | --- | --- |
| - Frequency of the following behaviors this week: | | | - In the past year, have you: | | |
| - physical distancing - cleaning and handwashing - following news media - checking social media - mask-wearing | | (0 = not at all; 4 = more than once daily) | - thought you had COVID-19 | | (no, yes) |
|  |  |  | - tested positive for COVID-19 | |  |
|  |  |  | - hospitalized for COVID-19 | |  |
|  |  |  | - ^1^Received COVID-19 vaccine first dose  (1 = yes, all doses; 2 = yes, another dose needed; 3 = no but planning to; 4 = no and not planning to; 5 = prefer not to say) | | |
| - Regional outbreak restrictions (closure of borders, schools, public places, non-essential stores or workplaces; no deliveries; physical distancing; stay at home; gatherings restricted, masking, none; other) (select all that apply) - Number of months spent isolating/distancing from people you do not live with (enter number) - Number of people currently living with (1 to 7+) | | |  |  |  |
|  |  |  | - Reason for vaccination decision (open text) | | |
|  |  |  | - **Willingness to receive COVID-19 booster dose in future (no, yes)** | | |
|  |  |  | - **Reason for response about future booster does  (open text)** | | |
| - Concern how the coronavirus might affect: | | | - Required to wear mask in public places (no, yes) | | |
| - you personally | | (0 = not at all;  4 = very) | - Would you wear a mask, even if not required  (no, yes) | | |
| - others | |  |  |  |  |
| - Degree to which days this week felt the same  (0 = very different; 4 = very similar) | | | - Reason for response about mask wearing  (open text) | | |
| - Beck Hopelessness Scale: Hopelessness | | | - Children under 18 received vaccine (no, yes, n/a) | | |
| - CESD-R: Loneliness during past week | | | - Reason for response about children vaccine (open text) | | |
| - **IUS-12: Intolerance of uncertainty** | | | - Willingness for children to receive booster  (no, yes, n/a) | | |
| - ^2^**PHQ-9: Severity of depressive symptoms** | | |  |  |  |
| - ^2^**GAD-7: Severity of anxiety symptoms** | | | - Reason for response about children booster (open text) | | |
| - ^†^Future Imagining Task (future, control) | | | - ^†3^Political affiliation with regard to: | | |
| - Extent thinking about present or future   (1=entirely present; 7=entirely future) | | | - social issues | 1= very left; 4=center;  7 = very right | |
|  |  |  | - economic issues |  |  |
| - Frequency of thinking about the imagined event   (1 = not at all; 7 = very often) | | | - ^†4^Indicate confidence in: | | |
|  |  |  | - science - scientists | (0 = Not at all;  10 = A lot) | |
| - Imagined event perspective (1 = own perspective; 7 = outside perspective) | | |  |  |  |
|  |  |  | - Agreement with statements about scientific knowledge: | | |
| - Imagined event detail   (1 = not much detail; 7 = a lot of detail) | | | - It is nothing but opinion | (0=Strongly disagree; 10=Strongly agree) | |
|  |  |  | - It can only be obtained through unbiased systematic research |  |  |
| - Imagined event emotionality  (1 = very negative; 7 = very positive) | | |  |  |  |
| - Imagined event importance   (1 = not at all; 7 = very important) | | | - How opposed or supportive is the media you engage with about: | | |
| - Imagined event likelihood  (1 = not at all; 7 = very likely) | | | - vaccines | 0=strongly opposed; 5=neither; 10=strongly supportive | |
|  |  |  | - scientific research |  |  |
| - When imagined event takes place (enter years) | | |  |  |  |
| - **Discounting of Future Rewards (AuC)** | | | - How balanced is the media you engage with about: | | |
| - Discounting of Future Losses (AuC) | | | - vaccines | 0=focuses only on weaknesses; 5=balanced;  10=focuses only on strengths | |
| - Anticipated frequency of behavior in coming weeks: | | | - scientific research |  |  |
| - physical distancing - mask-wearing - cleaning and handwashing | (0 = not at all;  4 = more than once daily | |  |  |  |

| **Time 1**  **(June – August, 2021)** | | | **Time 2**  **(June – August, 2022)** |
| --- | --- | --- | --- |
| - **Age** | | |  |
| - **Gender** | | |  |
| - **Current location (country, region)** | | |  |
| - **Highest level education attained** | | |  |
| - Employment status (employed, unemployed, retired) | | |  |
| - Current/most recent occupation (ISCO-08 categories) | | |  |
| - **Essential worker (no, yes)** | | |  |
| - Employment change due to pandemic (no change, got/lost job, more/fewer hours, work from home, changed jobs, on leave, retired) (select all that apply) | | |  |
| - **Indicate personal income on sliding scale relative to other incomes in the same region** | | |  |
| - **current** | **(0=low; 50=average; 100=high)** | |  |
| - before the pandemic |  |  |  |
| - In the past year have you: | | |  |
| - thought you had COVID-19 | | (no, yes) |  |
| - tested positive for COVID-19 | |  |  |
| - hospitalized for COVID-19 | |  |  |
| - ^1^Received COVID-19 main dose  (1 = yes, all doses; 2 = yes, another doses needed; 3 = no but planning to; 4 = no and not planning to; 5 = prefer not to say) | | |  |
| - Overall present health self-rating (0=excellent; 4=poor) | | |  |
| - Do you have: | | |  |
| - Conditions affecting heart, lungs, liver, or kidneys - Immunodeficiency disorder  (HIV/AIDS, leukemia) - Hypertension or high blood pressure - Diabetes or high blood sugar - Current smoker or vaper - Cancer requiring chemotherapy/radiation - Accident or condition affecting brain | | (no, yes) |  |
| - Anxiety that interferes with functioning - Major depressive disorder diagnosis - Ongoing psychiatric illness - Regular use of recreational drugs - Gambling problems | |  |  |
| - Average number of alcoholic beverages weekly (enter number) | | |  |

*Note.* Response options/format are provided in parentheses. Variables included in the main analyses of the current study are in bold. ^†^Additional control variables included in supplemental analysis (Table S2); ^1^Responses collapsed into binary variable (yes= 1,2; no= 3,4) for analysis. ^2^Total scores summed to create *Psychological Distress* variable; ^3^ Responses from items averaged to form *Political Affiliation* variable; ^4^ Responses from items averaged to form *Trust in Science* variable; AuC=Area-under-the-Curve; CESD-R = Centre for Epidemiological Studies Depression Scale – Revised; GAD = General Anxiety Disorder; ISCO = International Standard Classification of Occupations; PHQ = Patient Health Questionnaire.

**S2. Supplemental Analysis**

In addition to testing the predicted model reported in the results, we also conducted analyses including additional control variables: trust in science and political affiliation (collected at Time 2), and the Future Thinking condition (at Time 1). Our results show that the effect of delay discounting on booster vaccine willingness remains significant, even after controlling for these additional variables.

**Table S2. Results of the multilevel logistic regression model predicting booster willingness**

| Fixed Effects | *b* | *SE* | *z* | *p* | OR | 95% CI |
| --- | --- | --- | --- | --- | --- | --- |
| Intercept | 1.89 | 0.393 | 4.82 | < .001 | 6.64 | [3.08, 14.34] |
| Age^†^ | 0.02 | 0.005 | 3.09 | .002 | 1.02 | [1.01, 1.03] |
| Gender | 0.02 | 0.117 | 0.18 | .857 | 1.02 | [0.81, 1.29] |
| Education level | 0.01 | 0.081 | 0.02 | .986 | 1.00 | [0.86, 1.17] |
| Relative income^†^ | -0.13 | 0.061 | -2.05 | .040 | 0.88 | [0.78, 0.99] |
| Essential worker status | -0.22 | 0.136 | -1.60 | .110 | 0.81 | [0.62, 1.05] |
| Psychological distress^†^ | 0.06 | 0.036 | 1.74 | .083 | 1.07 | [0.99, 1.14] |
| Intolerance of uncertainty^†^ | -0.07 | 0.066 | -1.10 | .271 | 0.93 | [0.82, 1.06] |
| Delay discounting (AuC) | 0.55 | 0.227 | 2.43 | .015 | 1.73 | [1.11, 2.71] |
| Trust in science^†^ | 0.67 | 0.056 | 11.89 | < .001 | 1.95 | [1.75, 2.17] |
| Political affiliation | -0.16 | 0.023 | -7.17 | < .001 | 0.85 | [0.81, 0.89] |
| Future thinking condition | 0.01 | 0.111 | 0.08 | 0.94 | 1.01 | [0.81, 1.26] |
| Random Effects | Estimate | *SD* |  |  |  |  |
| Intercept error variance (country) | 0.15 | 0.39 |  |  |  |  |

*Note*. ^†^ The variable was scaled to improve model fit. AuC=Area-under-the-Curve. CI = Confidence interval; OR=odds ratio; SD=standard deviation; SE=standard error of the mean. Female gender, non-essential worker status, and control condition were used as reference categories in the analysis.
